# Topical Application of Peptide Nucleic Acid Antisense Oligonucleotide for MMP-1 and Its Potential Anti-Aging Properties

**DOI:** 10.3390/jcm12072472

**Published:** 2023-03-24

**Authors:** Young In Lee, Sang Gyu Lee, Inhee Jung, Jangmi Suk, Chaemin Baeg, Seon-Young Han, Jeong Yeon Seo, Daram Jung, Yeasel Jeon, Ju Hee Lee

**Affiliations:** 1 Department of Dermatology & Cutaneous Biology Research Institute, Yonsei University College of Medicine, Seoul 03722, Republic of Korea; 2 Scar Laser and Plastic Surgery Center, Yonsei Cancer Hospital, Seoul 03722, Republic of Korea; 3 Global Medical Research Center, Seoul 06526, Republic of Korea; 4 OliPass Corporation, Yongin-si 17015, Republic of Korea

**Keywords:** anti-aging, peptide nucleic acid, matrix metalloproteinase-1, *PNA-20 CEF*, collagen I

## Abstract

Matrix metalloproteinase-1 (MMP-1) is a zinc-containing endopeptidase that degrades dermal collagen and other extracellular matrix molecules. It is recognized as one of the most important indicators of cellular senescence and age-related skin changes. Here, we introduced a novel MMP-1 peptide nucleic acid (PNA) derivative—*PNA-20 carboxyethyl fluorene (CEF)*—which can interact with and consequently silence the MMP-1 gene sequence. The investigation on the efficacy of *PNA-20 CEF* in MMP-1 silencing in human dermal fibroblasts revealed significantly decreased expression of MMP-1 at both gene and protein levels. Treatment with *PNA-20 CEF* showed significantly increased expression of collagen I protein, indicating its potential role in preventing the degradation of collagen I and consequently combating the skin aging process. Its topical application on 3D human skin tissue showed successful absorption into the epidermis and the upper dermis. Furthermore, the additional 4-week single-arm prospective study on 21 Asian women revealed improvements in facial wrinkles, skin moisture, elasticity, and density after the use of the topical *PNA-20 CEF* cosmeceutical formulation. Additional in-vitro and ex-vivo studies are needed for a comprehensive understanding of the skin anti-aging effects of MMP-1 PNA.

## 1. Introduction

Aging of the skin is a complicated process that occurs because of both natural intrinsic and extrinsic aging processes. With age, the ability of the human body, including that of the skin, to resolve inflammation is markedly reduced, resulting in an imbalance between pro- and anti-inflammation. This causes a chronic low-grade pro-inflammatory status known as “inflammaging”, eventually leading to cellular aging [1]. Among the extrinsic factors aggravating cellular aging, ultraviolet radiation (UVR) exposure produces one of the most powerful effects. Repetitive UV irradiation induces large-scale deletions in mitochondrial DNA, resulting in dysfunction and aging owing to reduced collagen synthesis and increased collagen breakdown [2,3]. Moreover, it alters collagen homeostasis itself by UVR-induced hyperactivity of matrix metalloproteinases (MMPs), including MMP-1 (interstitial collagenase), MMP-3 (stromelysin), and MMP-9 (gelatinase), because of the oxidative stress-mediated activity of activator protein-1 (AP-1) and nuclear factor-kappa beta (NF-κB) transcription factors [4]. 

As the average human life expectancy increases, there is increasing interest globally in the anti-aging field, resulting in the establishment of the aging-related nutraceutical market, which has become one of the largest consumer markets [3]. Among various anti-aging products, topical retinoids are one of the most used active ingredients. Retinoids can promote keratinocyte proliferation and collagen synthesis by inhibiting collagen degradation and MMP activities [5]. Ascorbic acid is a water-soluble molecule that is also common in anti-aging cosmeceutical products; it functions as an antioxidant, cofactor in collagen synthesis, and tyrosinase inhibitor [6]. Hydrolyzed collagen tripeptide is another example of an active anti-aging ingredient which acts by increasing collagen expression levels while decreasing the expression of MMP-1, -3, and -9 [7].

As indicated by the abovementioned research, increasing the expression of collagen by decreasing MMPs is one of the core skin anti-aging tactics. MMP-1 is a zinc-containing endopeptidase that degrades dermal collagen and other extracellular matrix molecules [8]. Collagen, accounting for 30% of the total protein in the human body, is a major component of connective tissue [9] and is mainly regulated by MMPs and their natural inhibitors, tissue inhibitor metalloproteinases. UV-irradiated fibroblasts express increased secretion of MMP-1 through mitogen-activated protein kinase and AP-1 activation [10]. Therefore, increased MMP-1 activity is an important indicator of cellular senescence and age-related skin changes [8]. Decreased levels of MMP-1 protein can prevent age-related skin changes by preventing collagen degradation, the fundamental skin element related to the appearance of wrinkles. 

The active ingredients in anti-aging skin creams currently available increase collagen production while reducing MMP-1 expression [11,12,13,14]. With the aim of improving skin anti-aging, this study introduces a novel form of a peptide nucleic acid (PNA) derivative, *PNA-20 Carboxyethyl fluorene (PNA-20 CEF)*, which complementarily targets human MMP-1 pre-mRNA. It comprises 14 monomers that are derivatives of “N-(2-aminoethyl) glycine”, and its sequence is Fethoc-**T***A***CTC***A***C***C*A**T***A***T***A***T**-NH_2_, in which the six underlined and italicized bases are modified nucleobases, and the eight bold-lettered bases are unmodified nucleobases. The modified nucleobases of cytosine, adenine, and guanine predictably and complementarily hybridize with guanine, thymine, and cytosine, respectively. The degradation of collagen composing the dermis layer, caused by decreased MMP-1, is an integral part of aging. Therefore, *PNA-20 CEF* is designed to target the exon-intron junction of MMP-1 and produce non-functional MMP-1 by inducing exon skipping [wo2009113828]. The production of non-functional MMP-1 should produce non-functional MMP-1 proteins, decrease the effect of MMP-1, and induce anti-aging effects. Thus, we investigated the efficacy of *PNA-20 CEF* in inhibiting the expression of MMP-1, thereby increasing collagen expression in vitro, and performed a prospective, single-arm study on 21 Asian women with aging faces to investigate the clinical anti-aging efficacy of a cosmeceutical-formulation of *PNA-20 CEF* by estimating rejuvenation-related biophysical skin parameters. 

## 2. Materials and Methods

### 2.1. Preparation of PNA-20 CEF

*PNA-20 CEF* was produced by the OliPass Corporation as a 14-mer peptide nucleic acid complementarily targeting a 14-mer RNA sequence in human MMP-1 pre-mRNA. In brief, *PNA-20 CEF* was synthesized via solid-phase peptide synthesis on an automatic peptide synthesizer by Fmoc chemistry based on the method disclosed in a patent disclosure with minor modifications [15]. H-Rink Amide ChemMatrix resin was purchased from PCAS BioMatrix Inc. (Quebec, Canada) and was used as a solid support. After synthesis, *PNA-20 CEF* was purified by C18-reverse phase high-performance liquid chromatography (HPLC; water/acetonitrile or water/methanol 0.1% trifluoroacetic acid) and identified using high-resolution mass spectrometry. Its sequence was confirmed by measuring melting temperature (Tm) against complementary DNAs. Detailed manufacturing processes are available on request.

### 2.2. Cell Culture 

A Human Dermal Fibroblast (HDF) cell line was obtained from American Type Culture Collection (ATCC^®^; Manassas, VA, USA) and cultured in Dulbecco’s modified Eagle’s Medium (Lonza, Walkersville, MD, USA) supplemented with 1% Penicillin-Streptavidin (Gibco, Grand Island, NY, USA) and 10 % fetal bovine serum (Gibco). The cell line was incubated at 37 ℃ in a humidified 5% CO_2_/95% air atmosphere. 

### 2.3. Enzyme-Linked Immunosorbent Assay (ELISA)

HDFs (5 × 10^4^ cells/well) were seeded in 6-well plates and treated with a 6-point concentration of *PNA-20 CEF* or 0.05% OliPass RNA RS.301 OLV cream when cells reached over 80% confluence. After a 24 h incubation, the culture medium was harvested for PIP1 α1 and MMP-1 detection. The Human Pro-Collagen Ⅰ α1 SimpleStep ELISA kit (ab210966; Abcam, Cambridge, MA, USA) and Human MMP-1 ELISA Kit (ab100603; Abcam) were used according to the manufacturer’s instructions.

### 2.4. Quantitative Reverse Transcription-PCR (qRT-PCR)

HDFs (5 × 10^4^ cells/well) were seeded in 6-well plates and treated with a 6-point concentration of *PNA-20 CEF* or 0.05% OliPass RNA RS.301 OLV cream when cells reached over 80% confluence. After a 24 h incubation, total RNA was obtained from the incubated cells using RNAiso Plus (Invitrogen, Waltham, MA, USA) and synthesized into cDNA using an RNA to cDNA EcoDry Premix Kit (Takara Sake, Berkley, CA, USA). The Taqman primer of MMP-1 (Hs00899658_m1; Applied Biosystems, Waltham, MA, USA), and Taqman Gene Expression Master Mix were used to perform qRT-PCR. The primers used here were shown to be stable by the manufacturer. For normalization of MMP-1 expression levels, GAPDH (Hs02786624_g1; Applied Biosystems) was used as the control, and these results were analyzed using the 2^−ΔΔCt^ method.

### 2.5. Western Blotting

To extract proteins from HDFs treated with a 6-point concentration of *PNA-20 CEF*, radioimmunoprecipitation assay (RIPA) buffer (Cell Signaling Technology, Danvers, MA, USA) was used. The protein concentration was measured using the bicinchoninic acid (BCA) protein assay kit (Thermo Fisher Scientific, IL, USA). A total of 25 µg/µL of protein from each treatment group was subjected to 8% sodium dodecyl sulfate-polyacrylamide gel electrophoresis (SDS-PAGE). The resolved proteins were transferred to a polyvinylidene fluoride (PVDF) membrane (0.45 µm; EMD Millipore, MA, USA). The membrane was blocked with 5% skim milk and incubated overnight at 4 °C with anti-MMP-1 (1:2000 dilution; sc-58377, Santa Cruz, TX, USA), anti-Collagen Ⅰ (1:2000 dilution; 1310-01, Southern Biotech, Birmingham, AL, USA), and anti-β-actin (1:100,000 dilution; A3845, Sigma Aldrich, St. Louis, MO, USA) antibodies. Then, each membrane was incubated with the corresponding secondary antibodies (goat anti-mouse, CST, Cat. No. 7076V; rabbit anti-goat, Santacruz, Cat. No. 2768) diluted by a three thousandth for 1 h at room temperature. The antigen–antibody complexes were visualized using Immobilon western chemiluminescent horseradish peroxidase (HRP) substrate (Millipore), and then further analyzed using LuminoGraph II EM (ATTO Technology, Inc., Amberst, NY, USA) and CS analyzer (version 4, ATTO).

### 2.6. Immunofluorescence Staining

The reconstructed human micro skin-tissue model, Neoderm^®^-ED, was obtained from Tego Science (Seoul, Korea) and incubated for 24 h in a humidified atmosphere containing 5% CO_2_ at 37 °C after covering with 1 μM FAM-labeled *PNA-20 CEF*. Following fixation with 4% paraformaldehyde, the samples were made into 20 µm-thick frozen sections. Subsequently, these sections were fixed using VECTASHIELD^®^ Antifade Mounting Medium with 4′, 6-diamidino-2-phenylindole (DAPI) (Sigma Aldrich, St. Louis, MO, USA). Transpermutation of FAM-labeled *PNA-20 CEF* into human skin tissue was determined using a confocal laser scanning microscope (LSM 900 with Airyscan2, ZEISS, Carl Zeiss, Heidelberg, Germany).

### 2.7. Participants

A total of 22 healthy females aged 40–50 years with distinct wrinkles around their eyes were recruited for this clinical study. The exclusion criteria were participants having a history of the following: infectious skin disease, allergies or hypersensitivity, lesions on the test area, adverse responses to medicines, cosmetics, or routine light exposure, using similar medicines as our product, and any form of dermatologic treatment (e.g., Botox, laser, fillers, scaling, and tattoos) up to 3 months before this study. All participants were instructed to apply OliPass RNA RS.301 OLV cream on their faces after washing twice daily for 4 weeks to assess for clinical efficacy and safety. One participant dropped out of because she could not follow the treatment schedule. This clinical study was approved by the Institutional Review Board (IRB) of the Global Medical Research Center (IRB number: GIRB-21N01-GU), performed in compliance with the ethical principles of the Declaration of Helsinki, and informed consent was obtained from each participant. The present clinical study was approved by the Korea Center for Disease Control and assigned a Clinical Research Information Service number (KCT0007742).

### 2.8. Evaluation of Clinical Efficacy

OliPass RNA RS.301 OLV cream was manufactured as a facial anti-aging cream formulation containing *PNA-20 CEF*. A 24 h pre-clinical cream patch test was performed on the upper back of healthy adult participants between 19 to 59 years of age. The OliPass RNA RS.301 OLV cream showed a skin irritation index of 0.0 and was therefore considered a non-irritating skin product.

After the pre-clinical study, participants in the prospective single-arm clinical study were instructed to apply OliPass RNA RS.301 OLV cream twice daily for 4 weeks. All study participants were investigated at baseline, 2 weeks, and 4 weeks after treatment initiation using the Ultrascan UC22 (Courage Khazaka Electronic GmbH, Köhn, Germany), a device used to measure epidermal and dermal density changes. Skin moisture changes were determined using Corneometer^®^ (Courage Khazaka Electronic GmbH, Köhn, Germany). The inner and surface elasticity of the skin were measured using the Dermal torque meter (DTM; Dia-Stron, Hampshire, UK) and Cutometer Dual MPA580 (Courage Khazaka Electronic GmbH, Köhn, Germany). For changes in the depth of periorbital wrinkles, the skin analysis camera system (Antera 3D CS; Antera 3D^®^, Miravex, Dublin, Ireland) was used. Each measurement was performed after participants were acclimatized to controlled environmental conditions (room temperature: 20–24 ℃, relative humidity: 45–55%) for 30 min whenever they were evaluated for clinical efficacy. All participants were asked to report any adverse events while using the OliPass RNA RS.301 OLV cream. The participants were surveyed regarding their satisfaction with the anti-aging efficacy of the cream using the following scale: 1 = unsatisfied, 2 = no change, 3 = slightly satisfied, 4 = satisfied, and 5 = very satisfied. The total cream ingredients are shown in Supplementary Table S2.

### 2.9. Statistical Analysis

All data were statistically analyzed for significance using the SPSS Package Program version 25 (IBM Corp., Armonk, NY, USA). Clinical data at baseline and at weeks 2 and 4 were compared using the repeated measures analysis of variance (ANOVA) or the Friedman test followed by the post-hoc Wilcoxon signed-rank test with Bonferroni correction according to the results from the normality test. Data are expressed as mean values ± standard deviation, and statistical significance was set at *p* < 0.05, *p* < 0.01, and *p* < 0.005. All experiments were independently conducted at least three times (n ≥ 3).

## 3. Results

### 3.1. In Vitro and 3D Skin Assessments of the Cellular Anti-Aging Effect of PNA-20 CEF

**Figure 1:**
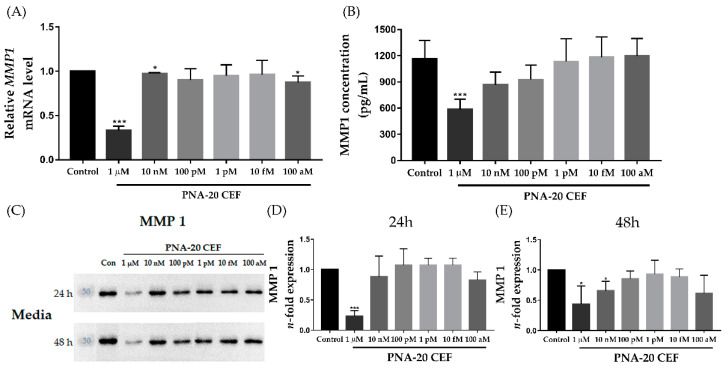
MMP-1 downregulation after *PNA-20 CEF* treatment of 1 μM, 10 nM, 100 pM, 1 pM, 10 fM, and 100 aM in HDFs. The mRNA level of *MMP-1* gene was reduced by treatment with 1 μM *PNA-20 CEF* for 24 h; qRT-PCR. (**A**). MMP-1 concentration measured using ELISA was also reduced after treatment with 1 μM *PNA-20 CEF* for 24 h (**B**). In the media obtained from cell cultures treated with *PNA-20 CEF*, the MMP-1 protein level measured via Western blot analysis was significantly reduced in the 1 μM *PNA-20 CEF* treatment for 24 h group (**C**,**D**). Treatment of 1 μM or 10 nM *PNA-20 CEF* for 48 h also significantly reduced MMP-1 protein expression (**E**). All experiments in the study were independently conducted at least three times (n ≥ 3). The results of the qPCR were normalized using GAPDH and the 2^−ΔΔCt^ method; * *p* < 0.05, *** *p* < 0.005 as obtained by the independent samples *t*-test, MMP; matrix metalloproteinase, HDF; human dermal fibroblast, qRT-PCR; quantitative reverse transcription-PCR, ELISA; enzyme-linked immunosorbent assay.

The relative *MMP-1* mRNA expression levels in human dermal fibroblasts (HDFs) were measured through quantitative reverse transcription-PCR (qRT-PCR). *MMP-1* mRNA expression levels of the control group and after treatment with 1 μM, 10 nM, 100 pM, 1 pM, 10 fM, and 100 aM of *PNA-20 CEF* were 1, 0.327 ± 0.021, 0.975 ± 0.004, 0.902 ± 0.054, 0.948 ± 0.051, 0.962 ± 0.065, and 0.873 ± 0.029, respectively (Figure 1A). *MMP-1* gene expression levels after treatment with 1 μM or 10 nM *PNA-20 CEF* were significantly reduced compared to those of the control group (* *p* < 0.05 and *** *p* < 0.005, respectively). We also performed a qRT-PCR to evaluate *MMP-1* expression levels when treated with *PNA-20 CEF* concentrations between 10 nM and 1 μM. The result showed that MMP-1 gene expression levels dose-dependently decreased as *PNA-20 CEF* concentrations increased; the MMP-1 expression levels reduced significantly when treated with 1 μM or 500 nM of *PNA-20 CEF* (Figure S1) (*** *p* < 0.005). MMP-1 concentration was also measured using an ELISA. MMP-1 concentrations of the control group and after treatment with 1 μM, 10 nM, 100 pM, 1 pM, 10 fM, and 100 aM of *PNA-20 CEF* were 1167.44 ± 106.31 pg/mL, 585.37 ± 58.82 pg/mL, 868.52 ± 72.29 pg/mL, 926.11 ± 84.01 pg/mL, 1132.41 ± 131.13 pg/mL, 1181.48 ± 116.93 pg/mL, and 1197.94 ± 100.35 pg/mL, respectively (Figure 1B). After 1 μM *PNA-20 CEF* treatment, MMP-1 concentration was significantly reduced compared to that of the control group (*** *p* < 0.005). Western blot analysis of MMP-1 from the HDF culture media after treatment with *PNA-20 CEF* for 24 h and 48 h is shown in Figure 1C. Treatment with 1 μM *PNA-20 CEF* for 24 h led to significantly reduced MMP-1 levels compared to that of the control group in the HDF cultured media (Figure 1D, *** *p* < 0.005). Treatment of 1 μM or 10 nM of *PNA-20 CEF* for 48 h also significantly reduced the MMP-1 level in the media (Figure 1E, * *p* < 0.05).

**Figure 2:**
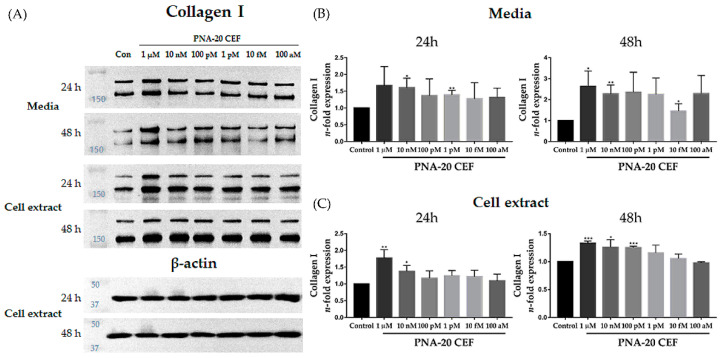
Collagen I protein expression after treatment with 1 μM, 10 nM, 100 pM, 1 pM, 10 fM, or 100 aM of *PNA-20 CEF* in HDFs; western blot analyses (**A**). In the media obtained from the cell culture treated with *PNA-20 CEF*, collagen Ⅰ was significantly induced in the 1 μM *PNA-20 CEF* treatment for 48 h group (**B**). Collagen Ⅰ was significantly induced in the 1 μM and 10 nM *PNA-20 CEF* treatment groups when the cell extract was treated for 24 h or 48 h (**C**). After treatment for 48 h, 100 pM *PNA-20 CEF* significantly induced collagen Ⅰ expression. All experiments were independently conducted at least three times (n ≥ 3); * *p* < 0.05, ** *p* < 0.01, *** *p* < 0.005 as obtained by independent samples *t*-test, HDF; human dermal fibroblast.

**Figure 3:**
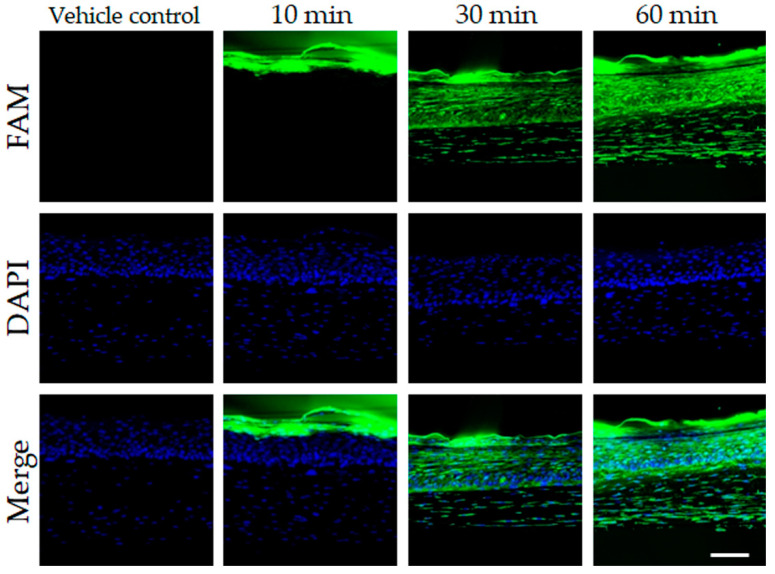
Visualization of *PNA-20 CEF* absorption in the reconstructed human 3D skin tissue using immunofluorescence staining. *PNA-20 CEF* showed increased absorption into the epidermis and the upper dermis with the passage of time. Scale bar indicates 100 μm.

Additionally, we investigated the effect of *PNA-20 CEF* on the expression of collagen I in fibroblasts via western blot analysis (Figure 2A). The media and cell extract samples used were obtained after treatment with 1 μM, 10 nM, 100 pM, 1 pM, 10 fM, or 100 aM of *PNA-20 CEF* for 24 h or 48 h. In the media, after 24 h treatment with 10 nM or 1 pM *PNA-20 CEF*, the collagen I protein expression increased significantly compared to that in the control group. After 48 h treatment with 1 μM, 10 nM, or 10 fM *PNA-20 CEF*, collagen I expression again increased significantly compared to that in the control group (Figure 2B, * *p* < 0.05, ** *p* < 0.01). These results indicated a reduction in collagen I degradation after *PNA-20 CEF* treatment. In the cell extract, the treatment with 1 μM or 10 nM *PNA-20 CEF* also showed significantly increased collagen I protein expression after 24 h or 48 h of the treatment (Figure 2C, * *p* < 0.05, ** *p* < 0.01, *** *p* < 0.005). Additionally, 100 pM *PNA-20 CEF* treatment showed increased collagen Ⅰ expression after 48 h compared to the control (*** *p* < 0.005). In the reconstructed human 3D skin tissue, 1 μM *PNA-20 CEF* was absorbed in the epidermis up to the upper dermis over time (Figure 3). Thus, topical application of *PNA-20 CEF* may be able to assist anti-aging of dermal fibroblasts.

### 3.2. In Vitro Assessment of the Cellular Anti-Aging Effect of OliPass RNA RS.301 OLV Cream

**Figure 4:**
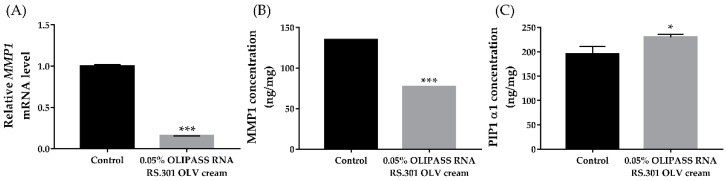
Levels of MMP-1 and collagen I before and after treatment with OliPass RNA RS.301 OLV cream in HDFs. Relative *MMP-1* mRNA expression levels obtained via qRT-PCR (**A**) and the concentration of MMP-1 obtained via ELISA (**B**) were reduced after treatment with 0.05% cream. The PIP1 α1 concentration was significantly increased by treatment with 0.05% cream compared with the control; ELISA. (**C**) All experiments in the study were independently conducted at least three times (n ≥ 3). The results of the qPCR were normalized using GAPDH and the 2^−ΔΔCt^ method; * *p* < 0.05, *** *p* < 0.005 by independent samples *t*-test, HDF; human dermal fibroblast; PIP1 α1, Pro-collagen Ⅰ α1; MMP; matrix metallopeptidase, qRT-PCR; quantitative reverse transcription-PCR, ELISA; enzyme-linked immunosorbent assay.

To evaluate the effect of OliPass RNA RS.301 OLV cream on the expression levels of MMP-1 and collagen in HDF cells, an additional qRT-PCR and ELISA were performed. The results indicate that the gene expression level of *MMP-1* after treatment with 0.05% cream (0.156 ± 0.001) was significantly reduced compared to that of the control group (1.000 ± 0.009) (Figure 4A, *** *p* < 0.005). Accordingly, the ELISA also revealed that the MMP-1 expression level after treatment with 0.05% cream (77.108 ± 0.001 ng/mg) was significantly reduced compared to that of the control group (134.852 ± 0.001 ng/mg; Figure 4B, *** *p* < 0.005), confirming its effect on silencing *MMP-1* gene expression and reducing MMP-1 expression. Meanwhile, an additional ELISA showed significantly increased expression levels of PIP1 α1 after treatment with 0.05% cream (230.169 ± 2.844 ng/mg) compared to that of the control group (195.60 ± 7.602 ng/mg; Figure 4C, * *p* < 0.05).

### 3.3. Participant Characteristics of the Clinical Trial

The clinical study was performed on 22 Asian female participants with distinct periorbital wrinkles. The mean age of the participants was 50.36 years (range, 41–59). The final analysis data were obtained from 21 participants as one participant was dropped because of noncompliance. The demographic data of participants are shown in Supplementary Table S1.

### 3.4. Clinical Efficacy of OliPass RNA RS.301 OLV Cream on Skin Aging 

**Figure 5:**
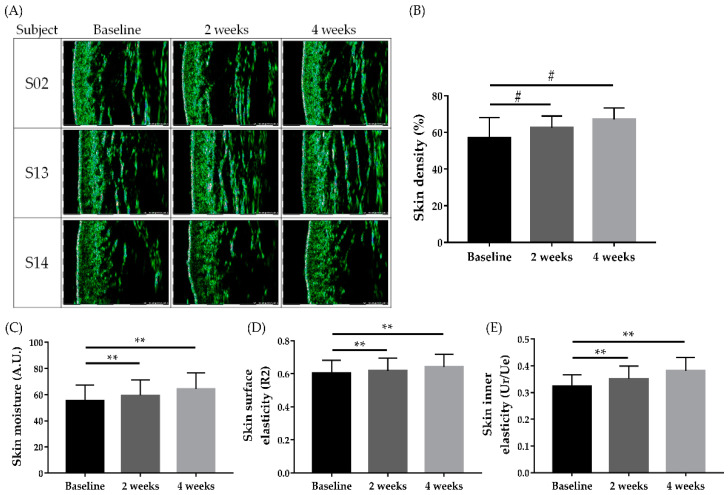
Changes in skin density, skin moisture, skin surface elasticity, and skin inner elasticity. Visualization of the epidermal and dermal densities was performed using Ultrascan UC22 (**A**). Skin density, skin moisture, skin surface elasticity, and skin inner elasticity, measured from the skin regions near the lateral canthus, were improved by applying the cream for 4 weeks (**B**–**E**); # *p* < 0025 (= 5%/2) by Friedman’s test and post-hoc Wilcoxon signed rank test with Bonferroni correction; ** *p* < 0.05 by repeated measurements of ANOVA.

**Figure 6:**
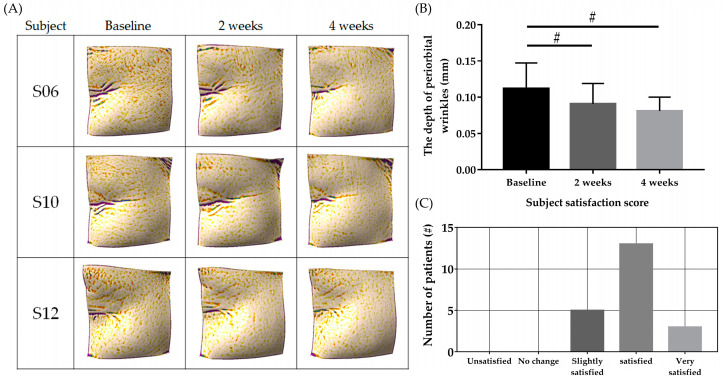
Changes in depth of periorbital wrinkles (**A**). Depth visualization was performed via the Antera 3D CS camera. The application of the cream for 4 weeks reduced the depth of periorbital wrinkles (**B**). Subjects who applied the cream for 4 weeks reported ‘Satisfied (‘very satisfied’: 3, ‘satisfied’: 13)’, while the remaining five participants reported ‘Slightly satisfied’ (**C**). No participants reported ‘Unsatisfied’ or ‘No change;’ # *p* < 0025 (=5%/2) as analyzed by Friedman test and post-hoc Wilcoxon signed rank test with Bonferroni correction.

The 21 participants were instructed to apply the cream to their entire face after washing twice daily for 4 weeks. OliPass RNA RS.301 OLV cream is produced as the facial cream formulation of *PNA-20 CEF*. The skin density measured using Ultrascan UC22 was significantly enhanced in week 2 (62.542 ± 3.205%) and week 4 (67.036 ± 3.212%) compared with the baseline (56.917 ± 5.599%; Figure 5A,B, # *p* < 0.05). The baseline skin moisture was 55.325 +/6.037 (A.U), significantly increasing to 59.368 ± 6.020 and 64.357 ± 6.130 in weeks 2 and 4, respectively (Figure 5C, ** *p* < 0.05). Meanwhile, the skin surface elasticity measured using a cutometer (R2) was also significantly increased in weeks 2 (0.619 ± 0.038) and 4 (0.641 ± 0.038) compared with the baseline (0.605 ± 0.039; Figure 5D, ** *p* < 0.05). The inner skin elasticity, as measured via a Dermal torque meter (Ur/Ue) was also significantly improved in weeks 2 (0.351 ± 0.024) and 4 (0.381 ± 0.025) compared with the baseline (0.323 ± 0.022; Figure 5E, ** *p* < 0.05). The improvement of periorbital wrinkles was visualized and analyzed using the Antera 3D CS camera (Figure 6A). Periorbital wrinkles were significantly reduced in weeks 2 (0.091 ± 0.014 mm) and 4 (0.081 ± 0.009 mm) compared with the baseline (0.112 ± 0.017 mm; Figure 6B, # *p* < 0.05). As a subjective clinical efficacy measurement, a satisfaction survey on clinical anti-aging efficacy after using the cream was performed after application of the test product for 4 weeks. Among the 21 participants, 16 (72.7%) reported ‘Satisfied (very satisfied: 3, satisfied: 13),’ while the remaining five reported ‘Slightly satisfied’ (Figure 6C). No participants reported ‘Unsatisfied’ or ‘No change.’

### 3.5. Assessment of Safety and Adverse Events

There were no reports from enrolled participants of serious adverse events, and none of the participants dropped out of the study because of adverse events, suggesting that the use of the cream was safe.

## 4. Discussion

Here, we constructed a novel MMP-1 oligonucleotide derivative, *PNA-20 CEF,* which can interact with the *MMP-1* gene sequence, specifically with nucleic acids, such as RNAs. The binding of an antisense RNA with sequences complementary to the target mRNA provides a powerful tool to modulate artificial gene expression, called RNA silencing [16]. Antisense oligonucleotides (ASO) can also bind to a pre-mRNA in the nucleus and affect the splicing of the pre-mRNA, producing an altered mRNA sequence [17,18]. However, one of the main barriers in applying ASO technology is the rapid degradation of DNA-based oligonucleotides in cells by nucleases [19].

Reactive oxygen species (ROS) produced by extrinsic aging factors, such as UVR exposure and tobacco smoking, have been known to overexpress MMP-1 [20,21]. MMP-1 has been identified as a skin aging-associated secreted protein (SAASP) which are proteins closely related to matrix degradation and pro-inflammatory processes [22]. Various antioxidants and food supplements with antioxidizing effects—Vitamin A (retinol), Vitamin C (ascorbic acid), Vitamin E (tocopherol), carotenoid, flavonoids, green tea, and selenium—have therefore been widely consumed for skin rejuvenation [23,24,25,26]. Considering the role of MMP-1 in skin aging, it is important to develop pharmaceuticals and cosmetics based on the mechanism of inhibiting MMP-1 activity.

A modified PNA is recognized as one of the most successful ASO derivatives. In PNA, the sugar-phosphate backbone of DNA or RNA is replaced with a pseudo-peptide backbone, while nearly identical geometry and spacing of the bases are retained. This modification can enhance the stability of oligonucleotides against enzymes that degrade DNAs, RNAs, and peptides [18]. It can also increase the PNA-RNA binding affinity, as RNA is negatively charged and PNA is electrically neutral [16]. Unlike small molecule drugs, however, the bioavailability of PNA is considerably limited because of its poor water solubility and cell permeability [27]. The *PNA-20 CEF* investigated here is a novel form of PNA established by introducing modified nucleobases with a cationic lipid or its equivalent covalently attached to them to overcome the cell permeability barriers.

Investigation of the efficacy of *PNA-20 CEF* in *MMP-1* silencing revealed significantly decreased mRNA expression levels of MMP-1 after treatment with *PNA-20 CEF* compared to that in the control in HDFs. Moreover, the results of the western blot analysis and ELISA showed decreased MMP-1 proteins detected from the cell internally and externally after treatment with *PNA-20 CEF*, confirming its action in inhibiting MMP-1 expression. The results suggest that treatment with *PNA-20 CEF* decreases the MMP-1 gene and protein expression in HDF cells internally and externally by producing non-functional MMP-1 as expected, which is dependent on dose efficacy and time. To further investigate the cellular anti-aging effect of *PNA-20 CEF,* collagen Ⅰ protein expression was analyzed via western blot analysis. The analysis showed a significant increase in collagen Ⅰ protein expression after *PNA-20 CEF* treatment compared to that in the control group. MMP-1 degrades and fragments collagen synthesized by the cells [28]. The increased collagen Ⅰ protein expression after *PNA-20 CEF* treatment was due to the MMP-1 silencing by *PNA-20 CEF.*

Aged dermal fibroblasts play a major role in dermal–epidermal interactions and overall skin tissue decline [29]. Previous research indicates that skin aging is primarily initiated at the dermis level, where macromolecular damage via extrinsic aging factors accumulates, causing resident fibroblasts to become senescent [7]. Therefore, a skin anti-aging pharmaceutical agent should enter the dermis level adequately. To confirm the enhanced efficacy of *PNA-20 CEF* in topical skin absorption after modification of its PNA molecular structure, we performed skin absorption tests on reconstructed human 3D skin tissue. The results revealed that *PNA-20 CEF* was effectively absorbed into the epidermis and the upper part of the dermis after OliPass RNA RS.301 OLV cream treatment. In addition, treatment with the cream in HDF cells reduced the MMP-1 mRNA expression level and protein production and increased the production of the PIP1 α1 protein. Hence, *PNA-20 CEF* inhibits the production of MMP-1 in epidermal keratinocytes and dermal fibroblasts, thereby increasing collagen production and leading to anti-aging clinical manifestations. Correspondingly, the 4-week single-arm prospective study revealed improvements in facial wrinkles, skin moisture, elasticity, and density without any adverse effects after the use of topical *PNA-20 CEF*. The satisfaction scores of the participants also reflected the overall improvement in aged skin. 

Our study had several limitations. First, there were a limited number of samples and a short-term follow-up period. A further prospective controlled study with a larger sample size and a longer follow-up period is required. A well-controlled study design should be constructed in the future to confirm the effect of *PNA-20 CEF* on MMP-1 silencing. Second, the in vitro study as well as the clinical study designs did not involve sufficient control data, such as scrambled PNA or a vehicle cream without the MMP-1 silencing effect. To test the penetration ability of *PNA-20 CEF* into the skin, we utilized a reconstructed skin model (Neoderm-ED). Although the skin equivalent is consistent with the epidermis and the dermis, its use to test penetration is still controversial [30]; therefore, an additional study with human ex vivo skin is needed to provide a better understanding of skin absorption. UV irradiation induces the synthesis and expression of MMP-1 by dermal fibroblasts, which is stimulated by the generation of excess ROS [8]. In vitro experiments with a UV-irradiated group as the positive control would have been instructive to evaluate the effect of *PNA-20 CEF* on MMP-1 and collagen I expressions. Furthermore, we only confirmed the anti-aging effect of *PNA-20 CEF* by observing collagen Ⅰ expression. As MMP-1 degrades both collagen I and III, an additional investigation on the expression level of collagen III would have been informative. Further in vitro and ex vivo studies to observe changes in other molecules that are well known as markers of cellular senescence, such as IL-6, p53, and p16, as well as β-galactosidase staining and tissue collagen density measurements, can be added to provide a comprehensive understanding of the effect of *PNA-20 CEF* targeting MMP-1 on skin anti-aging. *PNA-20 CEF* was designed only to target the exon–intron junction of MMP-1 to produce non-functional MMP-1 by inducing exon skipping. Future investigations into the effects of *PNA-20 CEF* that can also inhibit the activities of other MMPs involved in photoaging, including MMP-2, -3, and -9, would be useful to generate additional products against skin aging.

## 5. Conclusions

Our study showed the anti-aging effects of a novel ASO derivative targeting the MMP-1 oligonucleotide derivative via both laboratory and clinical studies. As many pharmaceutical and cosmeceutical agents with antioxidizing effects have been developed to treat skin aging with only limited efficacy, an innovative method targeting the mechanism of skin aging is appealing. Although the MMP-1 small interfering RNA (siRNA) is already reported in the literature to inhibit the expression of MMP-1 mRNA and protein levels in vitro [31], siRNAs are not only very expensive to manufacture but also possess poor cellular permeability. Moreover, the common carrier to transfer siRNAs to cells or the skin, lipofectamine, can induce cellular toxicity. Therefore, developing a novel agent, such as *PNA-20 CEF* with high biocompatibility to inhibit MMP-1 expression efficiently is critical. 

## Data Availability

The data employed and/or studied in this research can be obtained from the corresponding author upon request.

## Supplementary Materials

The following supporting information can be downloaded at: https://www.mdpi.com/article/10.3390/jcm12072472/s1, Table S1: Participant demographic data; Table S2: Total ingredients of OliPass RNA RS.301 OLV cream; Figure S1: Relative mRNA expression levels of *MMP1* in the HDF cells. The *MMP1* gene expression levels decreased dose-dependently as PNA-20 CEF concentration (*** *p* < 0.005).
