# Supplementary material for: Topical Application of Peptide Nucleic Acid Antisense Oligonucleotide for MMP-1 and Its Potential Anti-Aging Properties
Source: J Clin Med. 2023 Mar 24;12(7):2472. doi: 10.3390/jcm12072472 (PMC10095221; doi:10.3390/jcm12072472)

1. Supplementary Table

Supplementary Table S1. Participant demographic data

Screening No.	Sex	Age
S01	F	41
S02	F	45
S03	F	57
S04	F	55
S05	F	49
S06	F	51
S07	F	50
S08	F	51
S09	F	56
S10	F	57
S11	F	48
S12	F	41
S13	F	55
S14	F	50
S15	F	44
S16	F	59
S17	F	51
S18	F	48
S19	F	50
S20	F	59
S21	F	54

Supplementary Table S2. Total ingredients of OliPass RNA RS.301 OLV cream

Ingredients		
Water	Laminaria japonica extract	Sorbitan isostearate
Glycerin	Eclipta prostrata leaf extract	Hydrolyzed hyaluronic acid
Stearyl/Behenyl dimer dilinoleate	Piper methysticum leaf/root/stem extract	Theobroma cacao (cocoa) seed extract
Diisostearyl malate	Dextrin	Cholesterol
Phytosqualane	Hydrogenated \lecithin	Ceramide NP
Dimethicone	Glyceryl stearate	Pna-20 Carboxyethyl fluorene
Pentaerythrityl tetraisostearate	Copernicia cerifera (carnauba) wax	Pna-26 Carboxyethyl fluorene*
Phytosteryl/Isostearyl/Cetyl/	Polyacrylate-13	Pna-2 Carboxyethyl fluorene*
2,3-Butanediol	Polyisobutene	1,2-Hexanediol
trehalose	Hydrolyzed collagen	Polysorbate 20
Niacinamide	Adenosine	Polysorbate 80
Myristyl myristate	Behenyl alcohol	Fragrance
Betaine	Fructooligosaccharides	Hydroxyacetophenone
Cetearyl alcohol	Disodium EDTA	Ethylhexylglycerin
Peg-40 stearate	Ammonium acryloyldimethyltaurate/ vp copolymer	Beta-Glucan

* PNA-26 CEF is an oligonucleotide that complementarily targets human Acetyl CoA carboxylase beta pre-mRNA, while PNA-2 CEF targets human SNAP25 pre-mRNA.

2. Supplementary Figure

Supplementary Figure S1. Relative mRNA expression levels of *MMP1* in the HDF cells. The *MMP1* gene expression levels decreased dose-dependently as PNA-20 CEF concentration (***p < 0.005)

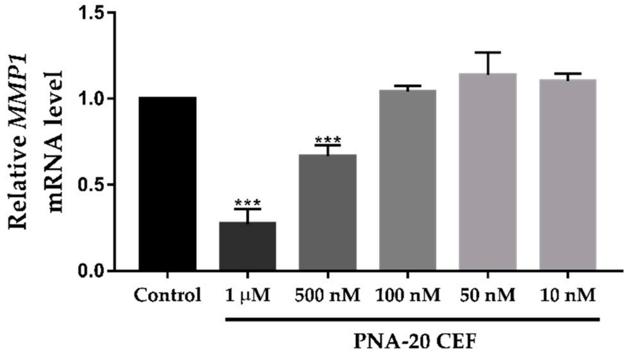

Supplement: Supplementary file 1 [file jcm-12-02472-s001.zip › jcm-2194690-SI.pdf]
